# Previously unrecognized and potentially consequential challenges facing Hsp90 inhibitors in cancer clinical trials

**DOI:** 10.1016/j.cstres.2024.08.002

**Published:** 2024-08-23

**Authors:** Cheng Chang, Xin Tang, David T. Woodley, Mei Chen, Wei Li

**Affiliations:** Department of Dermatology and USC-Norris Comprehensive Cancer Center, University of Southern California Keck Medical Center, Los Angeles, CA 90033, USA

**Keywords:** Human organs, Hsp90 chaperones, Hsp90 inhibitors, Clinical trials, Cancer

## Abstract

Targeting the heat shock protein-90 (Hsp90) chaperone machinery in various cancers with 200 monotherapy or combined-therapy clinical trials since 1999 has not yielded any success of food and drug administration approval. Blames for the failures were unanimously directed at the Hsp90 inhibitors or tumors or both. However, analyses of recent cellular and genetic studies together with the Hsp90 data from the Human Protein Atlas database suggest that the vast variations in Hsp90 expression among different organs in patients might have been the actual cause. It is evident now that Hsp90β is the root of dose-limiting toxicity (DLT), whereas Hsp90α is a buffer of penetrated Hsp90 inhibitors. The more Hsp90α, the safer Hsp90β, and the lower DLT are for the host. Unfortunately, the dramatic variations of Hsp90, from total absence in the eye, muscle, pancreas, and heart to abundance in reproduction organs, lung, liver, and gastrointestinal track, would cause the selection of any fair toxicity biomarker and an effective maximum tolerable dose (MTD) of Hsp90 inhibitor extremely challenging. In theory, a safe MTD for the organs with high Hsp90 could harm the organs with low Hsp90. In reverse, a safe MTD for organs with low or undetectable Hsp90 would have little impact on the tumors, whose cells exhibit average 3–7% Hsp90 over the average 2–3% Hsp90 in normal cells. Moreover, not all tumor cell lines tested follow the “inhibitor binding-client protein degradation” paradigm. It is likely why the oral Hsp90 inhibitor TAS-16 (Pimitespib), which bypasses blood circulation and other organs, showed some beneficiary efficacy by conveniently hitting tumors along the gastrointestinal track. The critical question is what the next step will be for the Hsp90 chaperone as a cancer therapeutic target.

## Highlights

• Hsp90b is the root of dose-limited toxicity.
• Hsp90a is a buffer of penetrated Hsp90 inhibitors.
• Vast variations in Hsp90 level in different human organs are real hurdles in trials.
• The “inhibitor binding - client protein degradation” paradigm is not universal.
• Targeting tumor-secreted Hsp90a (eHsp90a) is a viable alternative.

## Introduction

The rare capacity of heat shock protein-90 (Hsp90) to maintain the stability and functionality of almost all the components of cytoplasmic signaling networks was once viewed as a long-sought-after drug target to combat tumor resistance to single-molecule-based therapeutics, which still make up the vast majority of more than 1000 US food and drug administration (FDA)-approved cancer treatments today.^1^ The initial excitement has since led to at least 90 monotherapy clinical trials on a wide range of human cancers with several generations of either natural or synthetic small molecule inhibitors that all bind to the N-terminal denosine triphosphate/Adenosine diphosphate binding site of Hsp90 proteins (see ClinicalTrials.gov). The majority of the trials ended at phase I or phase II, and some completed phase III. To date, not a single Hsp90 inhibitor has received final approval from the US FDA for treatment of human cancers and targeting Hsp90 chaperones in cancer remains a work in progress^2^, ^3^, ^4^, ^5^, ^6^, ^7^, ^8^, ^9^, ^10^, ^11^, ^12^ (note: the approval of Hsp90 inhibitor Pimitespib/TAS-116 as an oral, instead of systemic, treatment of reoccurred and late-stage gastrointestinal stromal tumor [GIST] by the Ministry of Health, Labor and Welfare of Japan in 2022^13^ will be specifically discussed in a later section of this article).

While success in new drug development depends upon the combined responses of the drug candidate, the targeted tumor, and the host, collectively referred to as the “druggable window,” the focus of the previous clinical trials with Hsp90 inhibitors has largely limited to either the chemistry of the inhibitors, such as solubility, stability, and specificity, or defined responses from targeted tumors, such as induction of heat shock factors-dependent transcription and post-translational modification of Hgsp90 and client proteins inside the tumor cells.^3^, ^8^ A possible explanation for such a seemingly intentional bias is the untested believe that all the organs in the human body share the same level of sensitivity or resistance to the tested drug candidate. This was evident from using limited cell types and enzymes, such as peripheral blood mononuclear cells (PBMCs) and liver enzymes, as biomarkers during the trials. As a result, dose-limiting toxicities (DLTs), in which the tested inhibitors caused unacceptable adverse events in certain tissues and organs of the host prior to any detectable effect on the tumor, posted a major barrier in previous clinical trials. Among the 90 monotherapy clinical trials with various Hsp90 inhibitors, the reported DLTs clearly challenged the scientific foundation supporting the clinical trials that higher accumulation and higher sensitivity of tumors toward Hsp90 inhibitors than normal human organs creates a drug window for obtaining an FDA-acceptable efficacy. In this review article, as nonstakeholders of Hsp90 inhibitor drug development, we adhere to unbiased interpretations of the mouse genetic, isoform-distinctive, protein-quantitating studies of Hsp90 for the past 15 years, together with the recent availability of the Hsp90 expression levels in human organs from the Human Protein Atlas.^14^ As a result, this analysis enabled us to identify two main previously unrecognized challenges that might have directly contributed to the failures of past clinical trials.

## The “nut and shell” theory to define the distinct roles of Hsp90β and Hsp90α in cells

The results of gene-knockout studies in both cultured cells and mice have undisputedly demonstrated that Hsp90β is essential for the very survival of cells and animals, whereas Hsp90α is dispensable in both cases.^15^ Therefore, although Hsp90 ATP-binding inhibitors bind nondiscriminately to both Hsp90β and Hsp90α, it is conceivable that the blockade of the Hsp90β, but not Hsp90α, function is a direct cause of the reported DLTs in clinical trials. Hsp90α, on the other hand, titrates the cell-penetrated inhibitors and provides a “shield” of protection for Hsp90β. This guardian function of Hsp90α was previously referred to as the stress-buffering effect of Hsp90 by Neckers and Workman.^5^ Since the Hsp90β expression remains less variable among cell lines and organs in both mice and humans, the higher the level of Hsp90α, the lower inhibitor-caused toxicity in cells and organs is.

### Hsp90α and Hsp90β gene knockout in fly and mice

Gene depletion studies in mice have provided direct and physiological evidence in mammals that Hsp90α and Hsp90β have distinct functions. Voss *et al.*^16^ generated Hsp90β mutant mice by gene trap insertion into the exon 9 of the gene and found that heterozygous Hsp90β mutant mice developed normally, suggesting that only a fraction of Hsp90β (with the presence of full Hsp90α) is sufficient to support mouse development. However, mouse embryos with homogenous Hsp90β deletion remained normal by E9.0/9.5 but died a day later, even in the presence of Hsp90α. In contrast, Didier’s group first reported that mice with C-terminal 36-amino acid-deleted Hsp90α, which prevents the mutant Hsp90α protein to form a dimer, had little phenotypic difference from their wild-type counterparts, except that the male Hsp90α mutant mice were defective in spermatogenesis even in the presence of Hsp90β.^17^ The defect in spermatogenesis is consistent with an earlier study in *Drosophila* that reduced level of Hsp82 (E(sev)3A) (Hsp90α) was associated with a defect in spermatogenesis.^18^ The reported phenotype of Hsp90α^−/−^ mice was reproduced by Udono’s group.^19^ More interestingly, the same laboratory showed that Hsp90α supports spermatogenesis throughout adult life in mice.^20^ A recent study from our laboratory provided a possible mechanism for why Hsp90α is specifically involved in spermatogenesis. Tang *et al.*^21^ showed that hypoxia-inducible factor-1alpha (HIF-1α) was detectable only in the testis of mice among other organs screened in the same experiment. CRISPR–cas9-mediated knockout of Hsp90α destabilized HIF-1α in the mouse testis. While most tissues in mammals keep oxygen pressure in circulation between 2% and 9%, the testis is known to have a constant oxygen pressure lower than 1.5%, as well as a temperature two degrees lower than the rest of the animal body.^22^ Accordingly, the low oxygen pressure causes a constitutive expression of HIF-1α in premeiotic cells of the mouse testis^23^, ^24^ and in human sperm.^25^ This is the first *in vivo* evidence that Hsp90α specifically chaperones HIF-1α, and this mechanism of action by Hsp90α could not be replaced by Hsp90β.

### Hsp90α and Hsp90β gene knockouts in cultured cells

A dozen of earlier studies compared Hsp90α and Hsp90β using various gene (partial) downregulation techniques and functional assays in cultured cells and reported conflicting results. The conclusions of the studies could not be considered definitive due to technical limitations, such as incompleteness of gene deletion or lack of side-by-side comparisons of Hsp90α *versus* Hsp90β in the same experiments.^26^, ^27^, ^28^, ^29^, ^30^, ^31^, ^32^, ^33^, ^34^ Zou *et al.*^35^ used CRISPR/Cas9 gene-editing technology to stop translation of Hsp90α and Hsp90β genes within the exon-3 in MDA-MB-231 breast cancer cell line. The authors reported success in obtaining Hsp90α-knockout cell clones but failure in recovering any Hsp90β-knockout cell clones after two required rounds of drug selections. Like Hsp90α gene knockout in mice, the Hsp90α gene knockout showed little defect on survival and doubling time of the cells. Surprisingly, the absence of Hsp90α specifically nullified the cancer cells’ ability to migrate and invade in the absence of serum *in vitro* and to form tumors in nude mice. More intriguingly, the lost abilities of migration and invasiveness *in vitro* and tumorigenicity *in vivo* could be fully rescued by exogenously added or injected human recombinant Hsp90α, but not Hsp90β, protein.^35^ Tang *et al.*^21^ recently confirmed these findings for Hsp90α and Hsp90β in mouse embryonic fibroblasts. In conclusion, Hsp90α cannot compensate for the absence of Hsp90β for supporting cell survival and, likewise, Hsp90β is unable to replace the extracellular function of Hsp90α to promote normal cell migration and tumor cell invasion and tumorigenesis.

### Hsp90α gene mutation in humans

Passarino *et al.*^36^ conducted genetic polymorphism studies of Hsp90α (HSPCAL4, 14q31.3) and Hsp90β (HSPCB, 6p12) genes in healthy humans. Among 73 Caucasians involved in the study, the authors reported the identification of a frameshift mutation at amino acid-245 in the fifth exon on one of two Hsp90α gene alleles, which would have led to an early termination of 50% Hsp90α protein translation in a healthy human.^36^ Another mutation, G488H, in the middle domain abolishes the dimerization of Hsp90α.^37^ No similar mutations were reported in the Hsp90β gene among the 73 humans. Whether or not the amino acid-245 mutation exists in both alleles of the Hsp90α gene remains unclear (personal communication with Dr Passarino). There had been no follow-up information pertinent to the current understanding of Hsp90α, such as gender, health, and fertility status of these humans.

## Dramatic variations in Hsp90 expression in different cell types, tissues, and organs

### Steady-state Hsp90β and highly variable Hsp90α in cell lines

Although the statement of “1–2% Hsp90 of total cellular proteins” has been widely used in literature for the past several decades, in fact, this number did not come from formal experimental measurements and is remote from accuracy. On records, the first quantitation of the cellular Hsp90 protein was established by Sahu *et al.*^38^ using a classical biochemistry approach. Their measurements revealed that Hsp90 accounts for 2–3% of the total cellular proteins among normal cells and 3–7% of the total cellular proteins in cancer cells. Consistently, Finka and Goloubinoff^39^ recently obtained high-throughput proteomic data from 11 immortalized human cell lines and found that Hsp90 takes up approximately 2.8% of the total protein mass of the cells. More surprisingly, a study involving 12 (8 tumors and 4 normal) cell lines by Tang *et al.*^40^ from our laboratory reported a much greater range of 1.7–9% Hsp90 of the total cellular proteins among noncancer cell lines and 3–7% among the tumor cell lines. If we take the general assumption that a given type of cell expresses 7000 different proteins, that is, one-third of its total 20,000 protein-coding genes in the human genome, the Hsp90 expression alone is at least several hundred times higher than the rest of 6999 cellular gene products. No study has investigated why it is necessary to have such as disproportional level of Hsp90 proteins in a cell.

### Steady-state Hsp90β and highly variable Hsp90α in mouse and human organ

**Fig. 1:**
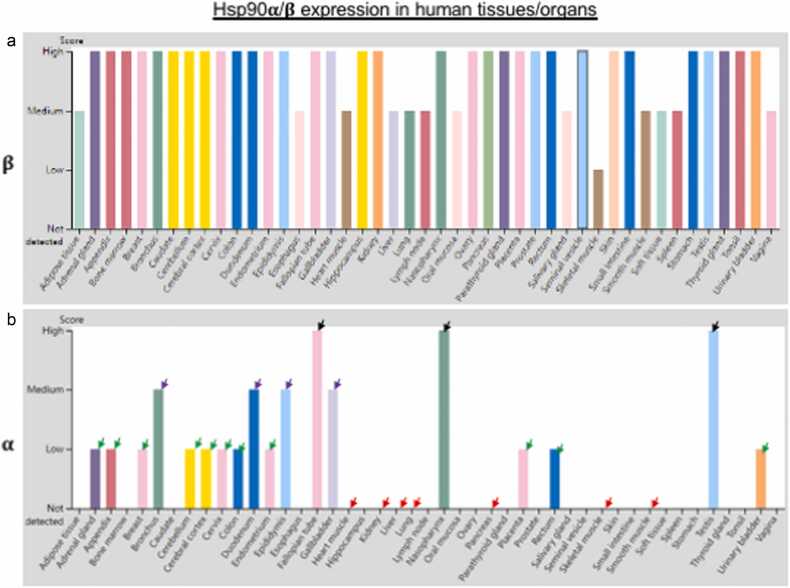
The relative expression levels of Hsp90β and Hsp90α in human tissues and organs. The variations in the relative expression of Hsp90β in human tissues and organs are small (a). The variations in the relative expression of Hsp90α in human tissues and organs are larger (b), ranging from undetectable (red arrows) to low (green arrows), to medium (purple arrows), and to high (black arrows). These data are consistent with the reported mouse organ data.^16^, ^17^, ^19^, ^21^ Taken from the Human Protein Atlas^14^ and modified.

Grad *et al.*^17^ showed that the Hsp90β protein level remains relatively constant, whereas Hsp90α protein level dramatically varies in different mouse organs, with high in testis and brain and low in heart, muscle, and pancreas. To minimize possible *in vitro* artifactual effects such as ischemic stress on protein levels on freshly excised mouse organs, Tang *et al.*^40^ developed a protocol to rapidly excise all organs from mice and immediately freeze them on dry ice with a minimum interval time. Equalized total protein extracts of different organs were separately on an SDS gel, Hsp90β, Hsp90α, and their combination was examined by Western immunoblot with anti-Hsp90 isoform-specific or pan antibodies. They showed that the Hsp90β protein levels remain less than two-fold difference, whereas the Hsp90α protein levels vary up to seven fold among different organs.^40^ The Hematoxylin and eosin data of Hsp90α and Hsp90β protein expression in human organs have recently become available from the Human Protein Atlas.^14^ As shown in Figure 1, similar to the reported data in mice, the Hsp90β protein levels among different human organs remain compatible. In contrast, the Hsp90α protein levels range from undetectable or extremely low in some organs, such as the heart, liver, and pancreas (red arrows), to high in others, such as the respiratory system, brain, and male/female organs (black arrows) and somewhere in between (green and purple arrows).

## There is no “common control cell” for toxicity in response to Hsp90 inhibitor treatment

The FDA’s guidelines for drug development are listed on its website briefly: (1) identification of a drug candidate, (2) *in vitro* and *in vivo* evaluations, (3) clinical trials, (4) drug review and approval, and (5) postmarket drug safety monitoring (https://www.fda.gov/patients/drug-development-process/step-2-preclinical-research). To our surprise, following literature and document search, the first Hsp90 inhibitor, 17-*N*-allylamino-17-demethoxygeldanamycin (17-AAG), entered cancer clinical trial in 1999, despite a lack of evidence for a clear druggable window between normal and tumor cells or between organs and tumors in animal models prior to the human trial approvals. The foundation of the trial appeared to be based on the finding that the HER2 receptor kinase, a prominent Hsp90 client, was a driver oncoprotein in certain breast tumors and showed an extreme sensitivity to 17-AAG.^41^, ^42^ In retrospect, one could not help wondering how the initial IND application had received approval from the US FDA. In fact, a number of publications that could have supported the approval of the trial came out after. These later supporting studies showed higher accumulation and/or higher sensitivity of tumor cells or tumor-bearing mice to Hsp90 inhibitors than normal cells and control mice. For instance, the widely cited publication by Kamal *et al.*^43^ reported a 100-fold difference in binding affinity of isolated cell-free Hsp90 protein complexes (note: not intact cells) from tumor cells than from control cells to 17-AAG. Using a similar cell-free protein binding assay, Chiosis’ group reported that normal cells could reach up to 700 to 3000-fold more resistance than tumor cells to purine-scaffold inhibitors.^44^, ^45^, ^46^, ^47^ The higher binding affinity by tumor cells was thought due to post-translational modifications in Hsp90 and oncogenic mutations in client proteins or both.^48^, ^49^, ^50^ However, data from intact cells in response to 17-AAG, 17-dimethylaminoethylamino-17-demethoxygeldanamycin (17-DMAG), and purine-based inhibitors were less conclusive. For instance, Premkumar *et al.*^51^ reported a rather moderate difference in cellular toxicity between 20% in normal cells and 50% in cancer cells to 17-AAG. Similarly, Lukasiewicz *et al.*^52^ showed 30–50% normal cell death *versus* 55–80% cancer cell death under treatment with 17-AAG or 17-DMAG. Chiosis’ group showed an approximately 20-fold difference in cell growth inhibition between tumor cells *versus* a normal cell line.^44^, ^45^, ^53^ Vilenchik *et al.*^47^ reported less than 10-fold difference in the IC50 values for inhibitor PU24FCI in cell growth inhibition between 2 normal and 15 cancer cell lines. Besides these *in vitro* studies, there were few animal studies on the critical issue of druggable windows before and even after the clinical trials. Three years after the start of the initial clinical trial, Solit *et al.*^54^ reported that the maximally tolerated dose of 17-AAG was higher in control mice than in tumor-bearing mice, albeit the concerns for its drug administration schedule-dependent and the poor health of the tumor-bearing mice in comparison to the control healthy mice. In addition, recent findings in cultured cells and in mouse and human organs indicate that there are no “common normal cell type or organ type controls” for all other cell types and organs due to the vast variations in Hsp90 levels in different types of cells and organs. Tang *et al.*^40^ recently compared randomly selected six tumor and four nontumor cell lines under identical experimental conditions for (1) relative expression levels of Hsp90 protein and (2) relative sensitivity to increasing dosages of 17-DMAG, a second generation, synthetic, water soluble, less off protein-bound, and more potent than 17-AAG.^55^ These authors showed that (1) cellular Hsp90 expression ranges from 1.7% to 9% of the total cellular proteins and (2) the cells with 1.7%–9% Hsp90 exhibited either extreme sensitivity or extreme resistance to 17-DMAG.^40^ Since the Hsp90β expression level remains compatible whereas the Hsp90α protein level varies dramatically among different types of cells and organs, it is conceivable that the variations in sensitivity or resistance among different types of the cells to 17-DMAG are likely due to a variable capacity of inhibitor titrating or buffering by Hsp90α.

## The “inhibitor binding > client protein degradation” paradigm does not hold up among different tumor cells

**Fig. 2:**
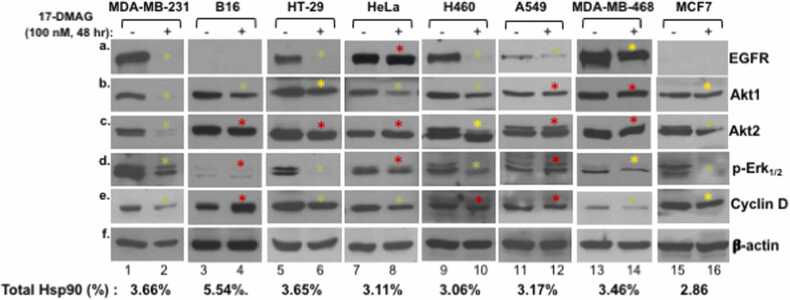
The paradigm of “Hsp90 inhibitor-causing client protein degradation” does not hold up in different tumor cell lines. Eight tumor cell lines were randomly selected, simultaneously cultured, and treated with 17-DMAG under identical conditions, as indicated. Total cellular proteins were equalized and analyzed by Western immunoblotting analysis with indicated antibodies. Comparisons in the band intensity of the same protein targets were only valid within the same cells without or with 17-DMAG treatment. The typical responses of MDA-MB-231 cells to Hsp900 inhibitors were used as the standard of the paradigm. Green arrow, complete degradation of clients; yellow arrow, partial degradation of clients; and red arrow, no/reverse response of clients. Abbreviation used: 17-DMAG, 17-dimethylaminoethylamino-17-demethoxygeldanamycin.

Source: This figure is taken with permission from a recent publication Tang *et al*.^40^

The foundation for supporting Hsp90 inhibitor clinical trials came from the landmark finding that 17-AAG directly binds to Hsp90 instead of its client protein v-src tyrosine kinase, resulting in dissociation of Hsp90 from v-src kinase and degradation of the v-src oncogene product in v-src-transformed mouse NIH3T3 and PC3 human prostate cancer cell lines.^56^ This discovery has since evolved to become the widely accepted paradigm for Hsp90’s mechanism of action and foundation for therapeutic development. Having repeatedly made inconsistent observations during experiments, Tang *et al.*^40^ decided to formally carry out side-by-side comparisons among eight different cancer cell lines using the client proteins from the so-called mitogenic pathway in response to the standardized treatment of 17-DMAG. In retrospect, this kind of screening study should have been thoroughly completed long before launching the clinical trials, but few previous publications did similar or larger scale of the comparisons. To the authors’ surprise, the responses of the different cancer cells were massively at odds with the paradigm for the Hsp90 inhibitors’ mechanism of action.^40^ As shown in Figure 2, the green dots represent the client proteins in indicated cancer cell lines that faithfully followed the paradigm; the yellow dots indicate the client proteins that partially followed the paradigm; and the (many) red dots that spread among different cancer cell lines point out the client proteins that did not follow the paradigm or exhibited opposite responses of the paradigm. In summary, (1) MDA-MB-231 was the only cell line in which all five selected client proteins perfectly followed the paradigm of Hsp90 inhibition; (2) even though MDA-MB-231 and MDA-MB-468 are both categorized as triple-negative breast cancer cells, the same client proteins responded differently; (3) epidermal growth factor receptor, the most sensitive client to Hsp90 inhibitors,^41^, ^42^ was reduced in five cell lines but remained unchanged in Hela cells; (4) the Akt1 level was reduced in four of the eight cancer cell lines, while it remained either partially reduced or unchanged in rest of the four cell lines; (5) downregulated clients, unchanged clients, and upregulated clients were all found within the same cells such as B16 and A549 cells in response to 17-DMAG; and (6) finally, these heterogeneous responses of the client proteins were unrelated to the total Hsp90 level (%) in each of the eight cell lines (as indicated underneath the western blot). A similar observation was previously made in single-cell lines by independent groups.^57^, ^58^ However, due to the immense importance of the issue, more extended studies with larger numbers of tumor cells must be carried out and thoroughly analyzed. While the cause of the heterogeneous responses from different tumor cell lines to Hsp90 inhibitors remains little beyond speculations, such as post-translational modifications and multiple oncogenic factors in the cells, the above observations could have posted serious challenges for the patient selection processes of the previous clinical trials.

## Occurrence of previously reported DLTs correlates with low Hsp90-expressing human organs

**Fig. 3:**
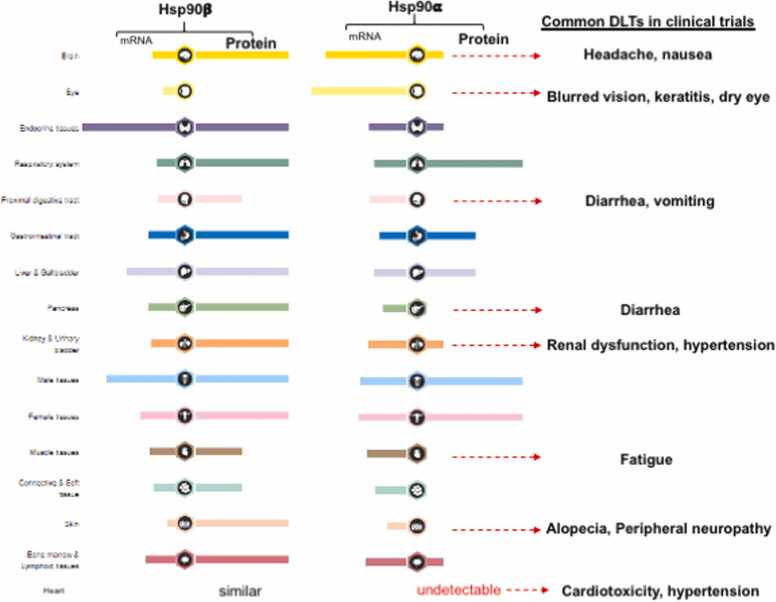
Reported DLTs all occurred in low Hsp90-expressing organs. Relative Hsp90β and Hsp90α (mRNA and protein) expression levels in various human organs are as indicated (taken from the Human Protein Atlas). The Hsp90β expression remains relatively constant, whereas the Hsp90α expression dramatically varies among different organs. The previously reported DLTs from 90 monotherapy clinical trials all occurred in the organs with undetectable or lower Hsp90α expression. Abbreviations used: DLT, dose-limiting toxicity; Hsp90, heat shock protein-90; mRNA, messenger RNA.

Since 2009, a dozen of excellent review articles has provided comprehensive analyses of drug related toxicities from some 90 phase I–II monotherapy cancer clinical trials with several generations of Hsp90 ATP-binding inhibitors.^1^, ^2^, ^3^, ^4^, ^5^, ^6^, ^7^, ^8^, ^9^, ^10^, ^11^, ^12^ The maximum tolerable dose (MTD) of the inhibitors was determined based on pharmacodynamic (PD) studies limited to liver toxicity and client protein measurements from isolated PBMC. The repeatedly reported DLTs from the inhibitor-treated patients of the previous clinical trials include ocular disfunctions, increased liver enzymes, fatigue, nausea, diarrhea, anorexia, renal dysfunction, dyspnea, and cardiotoxicity. These DLTs correlate with compromised functions of eye, liver, muscles, kidney, lung, pancreas, and heart. Based on the above information, we attempted to match the DLTs with the reported levels of Hsp90 expression in the corresponding human tissues and organs, using the Hsp90 protein data from Human Protein Atlas.^13^ The schematic representation of the attempted correlations is shown in Figure 3. The variation in Hsp90β expression among different human organs is relatively small (left color column). In contrast, the Hsp90α levels drastically vary (right color column) from undetectable such as the eye, heart, pancreas, connective tissues, skin, and muscles; low such as brain, endocrine tissues, digestive track, kidney, and bone marrow; medium such as gastrointestinal track and liver; and high such as lung and sex organs. It is clearly shown that the reported DLTs from previous clinical trials correlate with the organs that show either undetectable or low Hsp90α (far right column).

## Has the host, but not tumors, failed Hsp90 inhibitors in previous clinical trials?

**Fig. 4:**
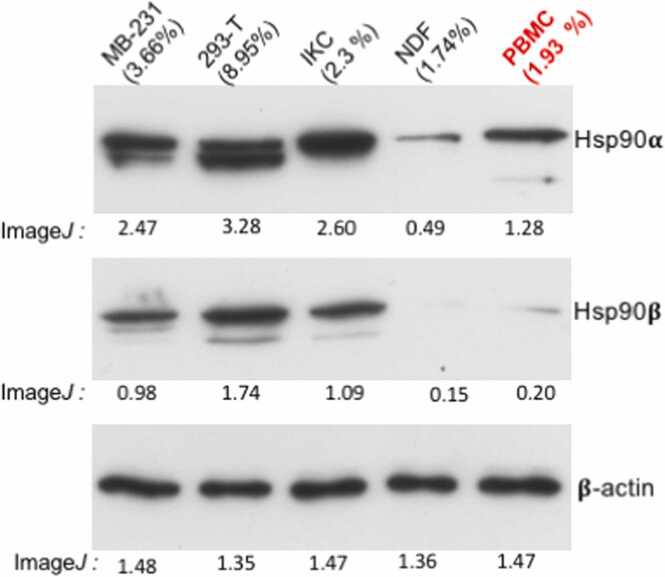
PBMC cannot be a common biomarker representing different cells, tissues, and organs. The percentage (%) of the total Hsp90 expression in PBMC was estimated by comparing to cell lines with previously known percentages of total Hsp90. The equalized total lysates of cells were resolved on the same SDS-PAGE, immunoblotted with indicated antibodies, and estimated based on data by Image*J*. Abbreviation used: PBMC, peripheral blood mononuclear cell; SDS-PAGE, sodium dodecyl sulfate–polyacrylamide gel electrophoresis.

Recent studies have come to the realization that the cells, tissues, and organs with lower levels of Hsp90 will be damaged first and more seriously by the inhibitors than the higher Hsp90α-expressing cells, tissues, and organs. Unfortunately, based on two unproven or less substantiated assumptions, (1) all human tissues and organs share similar Hsp90 expression and sensitivity to Hsp90 inhibitors, and (2) tumors are more sensitive to Hsp90 inhibitors than (all) human tissues and organs, previous clinical trials used a single-cell type, PBMC, for PD evaluations to determine the MTD of inhibitors. Noninvasive techniques, such as Positron emission tomography imaging, to monitor tumor drug responses could have been useful but were still a work in progress.^2^ Out of curiosity, we have recently compared the relative amount of Hsp90α and Hsp90β in PBMC with cell lines whose percentages (%) of Hsp90 in total cellular proteins have previously been established.^38^, ^39^, ^40^ As shown in Figure 4, the Hsp90α and Hsp90β levels in PBMC belong to the average level of Hsp90α and Hsp90β in the cell lines. If we extrapolate the clinical implication of the observation, previous PD studies using PBMC for MTD could not have predicted potential DLTs in Hsp90-undetectable organs, such as the eye, mussels, and heart. It is hard to imagine that the level of sensitivity of any tumor cells, which often show much higher Hsp90 expression than most normal cells, to Hsp90 inhibitors is still higher than human organs that show undetectable levels of Hsp90.

### From the point of the host system

It would have had been challenging, if not impossible, for previous clinical trials to conduct any single toxicity study “fair” to all human organs and establish an effective MTD of the Hsp90 inhibitor on tumors. A dosage of an inhibitor tolerable to low Hsp90-expressing organs, such as the eye, smooth muscle, and heart, would unlikely have clinically significant antitumor efficacy. In reverse, a dosage of an inhibitor tolerable to high Hsp90-expressing organs, such as blood cells, lung, and reproduction organs, may show significant efficacy on tumors, but it would most certainly damage the eye, muscle, pancreas, and heart. For instance, one of the most frequently reported DLT during previous clinical trials was fatigue. If the skeletal and smooth muscles become weakened by the inhibitor, it will not only reduce body strength but also weaken the heart to efficiently pump oxygen-rich and nutrient-rich blood to distant organs. Even the stomach will lose the ability to process the intake of food. In addition, inhibition of the hemeprotein maturation in cells would greatly compromise oxygen transport, causing fatigue and more.^59^ Solit and Chiosis^60^ were puzzled by their finding that an MTD of 17-AAG in their phase II trial in patients with metastatic melanoma showed little clinical efficacy. Their, however, PD study of the tumor biopsy samples taken before and after inhibitor treatment revealed little Hsp90 inhibition.^60^ With the new theories above, now it is easy to understand the puzzle.

### From the point of the tumors in the host

Most tumor cell lines analyzed so far express Hsp90 3–7% of the total cellular proteins, which is higher than the 2–3% Hsp90 reported in normal cell lines.^38^, ^39^, ^40^ It is understandable that tumor cells need higher levels of protection of their oncogenic driver genes than their cellular counterparts. Based on chaperone protection or “buffering effect’s point of view,”^4^ the above numbers already suggest that tumor cells have high degrees of protection than normal cells from the attack of inhibitors. While some believe the argument that tumor cells have more Hsp90 and are also more sensitive to inhibitors than normal cells (which sounds contradictory to begin with), it would be hard to imagine that a tumor-damaging dose of an inhibitor could spare the Hsp90-negative human organs such eye, pancreas, and heart. Therefore, once a dosage of the inhibitor is capable of damaging oncogenic drivers in tumor cells, it most certainly will harm its cellular counterparts in those Hsp90-less normal cells before tumor cells. In addition to discrepancy on inhibitor dosages, a recent report showed there is a great deal of heterogeneousness among different tumor cell lines and even the same type of tumors, such as breast and prostate cancers in response to a Hsp90 inhibitor.^40^ The finding of the study suggests that there might be a need to prescreen the patients of clinical trials to recruit only those “right group” of patients who share the same response of inhibitor binding > client protein degradation paradigm. Logistically, it would be unimaginable if each cancer patient had to be biopsied and biochemically predetermined for the mechanism of action by the to-be-trialed Hsp90 inhibitor. In fact, no one knows if the same clinically defined tumors in different patients share a similar Hsp90 expression and similar response to an Hsp90 inhibitor. That was likely the reason for Neckers and Workman^5^ to suggest stratification of cancer patients based on Hsp90 expression and oncogenic clients in the tumor cells prior to clinical trials. In brief, (1) the wide range of Hsp90 expression in normal organs and (2) the heterogeneous responses of tumors from different patients to the same inhibitor could have been a real cause for the failures of previous clinical trials.

## Does oral administration of Pimitespib/TAS-116 unintentionally kill two birds with one stone?

Administrations of Hsp90 inhibitors *via* circulation, including tanespimycin (17-AAG), retaspimycin hydrochloride (IPI-504), ganetespib (STA-9090), BIIB021 (CNF 2024), luminespib (NVP-AUY922), and onalespib (AT13387) have all caused similar DLTs in patients with various cancers, including advanced GISTs and as result none advanced to US FDA approval, despite some trial reached phase III clinical evaluation.^61^ The oral-administered Pimitespib (TAS-116, tablets 40 mg), also an N-terminal inhibitor of both Hsp90 isoforms by Taiho Pharmaceutical (Japan), was approved by Japan’s Ministry of Health, Labor and Welfare for treatment of advanced GIST, after the tumors have progressed and no longer respond to previously US FDA-approved protein tyrosine kinase inhibitor drugs (imatinib, sunitinib, and regorafenib) in 2022.^13^^,^^62^ Pimitespib has no permission for clinical applications in the United States, without clinical trials and approval by US FDA. It is noticed that Pimitespib initially entered phase I clinical trials in Japan and the UK with patients having a variety of solid tumors. However, its phase II and phase III trials exclusively focused on patients with GIST because of limited efficacy on tumors of other internal organs.^62^ GISTs are a type of cancer found in the gastrointestinal tract, in which 60% develop in the stomach and the rest of the 35% in the small intestine. Again, the level of Hsp70 expression in PBMC cells was used as the biomarker of the trials, since Pimitespib was reported not to bind Hsp70 in cytoplasm, GRP94 in endoplasmic reticulum, and TRAP1 in mitochondrial.^63^ Taken together, we speculate that the oral administration of Pimitespib would greatly reduce its penetration and spread to other internal organs and tumors other than the GI track and GISTs. Intuitively, oral Pimitespib directly flows into and hits the tumors in the GI track without barriers. It is conceivable that such a “localized” treatment would dramatically reduce the DLTs associated with systemic administration. Finally, it cannot be excluded that the reported efficacy of Pimitespib might result from its binding and inhibition of the tumor-secreted Hsp90α (eHsp90α) in GI track.

## Conclusions

High DLT and insignificant efficacy ended previous cancer clinical trials early and prevented the Hsp90 chaperone inhibitors from receiving US FDA approval in the past. To analyze the cause of the failures, we raise two previously unrecognized challenges from the host and tumors. First, the vast variations from undetectable-to-abundant Hsp90 expression in different human organs could make selection of fair biomarkers and identification of effective MTD difficult. The previously reported DLTs all come from low Hsp90-expressing human tissues or organs. It is hard to believe that often higher Hsp90-expressing tumors are still “more sensitive” to Hsp90 inhibitors than Hsp90-negative human organs, such as the eye, pancreas, and heart cells. Second, a recent preliminary finding that not all tumor cells follow the paradigm of “inhibitor binding > client degradation” could significantly complicate recruitments and responses of cancer patients in clinical trials.

## Funding and support

The authors have neither financial nor nonfinancial conflicts of interest. This work is supported by 10.13039/100000002National Institutes of Health grants GM067100 (to W.L.) and grants W81XWH-1810558 from the Congressionally Directed Medical Research Program (to M.C.).

## Author contributions

**Xin Tang:** Writing – original draft, Formal analysis, Data curation. **Cheng Chang:** Validation, Data curation. **Wei Li:** Writing – review & editing, Writing – original draft, Supervision, Resources, Data curation, Conceptualization. **Mei Chen:** Writing – original draft, Resources, Funding acquisition. **David T. Woodley:** Writing – review & editing.

## Declarations of interest

The authors declare the following financial interests/personal relationships, which may be considered as potential competing interests. Wei Li reports financial support was provided by the National Institutes of Health. Mei Chen reports financial support was provided by the National Institutes of Health. If there are other authors, they declare that they have no known competing financial interests or personal relationships that could have appeared to influence the work reported in this paper.

## Data Availability

Data will be made available on request.
